# A Preparatory Study for a Randomized Controlled Trial of Dietary Fiber Intake During Adult Pelvic Radiotherapy

**DOI:** 10.3389/fnut.2021.756485

**Published:** 2021-12-07

**Authors:** Rebecca Ahlin, Karin Bergmark, Cecilia Bull, Sravani Devarakonda, Rikard Landberg, Ida Sigvardsson, Fei Sjöberg, Viktor Skokic, Gunnar Steineck, Maria Hedelin

**Affiliations:** ^1^Division of Clinical Cancer Epidemiology, Department of Oncology, Institute of Clinical Sciences, Sahlgrenska Academy at the University of Gothenburg, Gothenburg, Sweden; ^2^Department of Biology and Biological Engineering, Chalmers University of Technology, Gothenburg, Sweden; ^3^Department of Infectious Diseases, Institute of Biomedicine, Sahlgrenska Academy at University of Gothenburg, Gothenburg, Sweden; ^4^Department of Molecular Medicine and Surgery, Karolinska Institutet, Stockholm, Sweden; ^5^Department of Pelvic Cancer, Karolinska University Hospital, Stockholm, Sweden; ^6^Regional Cancer Center West, Sahlgrenska University Hospital, Gothenburg, Sweden

**Keywords:** dietary fiber, psyllium husk, mobile phone application, gynecological cancer, pelvic radiotherapy

## Abstract

**Background:** Patients undergoing pelvic radiotherapy are often advised to omit fiber-rich foods from their diet to reduce the adverse effects of treatment. Scientific evidence supporting this recommendation is lacking, and recent studies on animals and humans have suggested that there is a beneficial effect of dietary fiber for the alleviation of symptoms. Randomized controlled studies on dietary fiber intake during pelvic radiotherapy of sufficient size and duration are needed. As preparation for such a large-scale study, we evaluated the feasibility, compliance, participation rate, and logistics and report our findings here in this preparatory study.

**Methods:** In this preparatory study of a fiber intervention trial, Swedish gynecological cancer patients scheduled for radiotherapy were recruited between January 2019 and August 2020. During the intervention, the participants filled out questionnaires and used an application. They also consumed a fiber supplement at first in powder form, later in capsules. Blood- and fecal samples were collected. The study is registered in clinicaltrials.gov (https://clinicaltrials.gov/ct2/show/NCT04534075?cond=fidura&draw=2&rank=1).

**Results:** Among 136 approached patients, 57 started the study and the participation rate for primary outcomes was 63% (third blood sample) and 65% (third questionnaire). Barely half of the participants provided fecal samples. Providing concise and relevant information to the patients at the right time was crucial in getting them to participate and stay in the study. The most common reasons for declining participation or dropping out were the expected burden of radiotherapy or acute side effects. Tailoring the ambition level to each patient concerning the collection of data beyond the primary endpoints was an important strategy to keep the dropout rate at an acceptable level. Using capsules rather than psyllium in powder form made it much easier to document intake and to create a control group. During the course of the preparatory study, we improved the logistics and for the last 12 participants included, the participation rate was 100% for the earliest primary outcome.

**Conclusion:** A variety of adjustments in this preparatory study resulted in an improved participation rate, which allowed us to set a final protocol and proceed with the main study.

## Introduction

The number of cancer survivors worldwide is steadily increasing and thereby the number of patients living with therapy-induced adverse effects (1, 2). Pelvic radiotherapy is a common treatment for cancer in the pelvic and lower abdomen (3). Epithelial cellular or microvascular injury resulting from treatment triggers intestinal inflammatory processes (4). Five symptom clusters (syndromes) related to intestinal health can appear years, or even decades, after completion of radiotherapy of a tumor in the pelvic cavity (5). The syndromes are related to an often recurring and urgent need to defecate, fecal leakage, leakage of mucus, uncontrollable flatulence, or anal bleeding (5). Radiation-induced survivorship diseases negatively affect the quality of life (6, 7).

There is currently insufficient high-quality evidence to recommend nutritional interventions during pelvic radiotherapy (8), and no uniform practice is adapted in health care. Clinical experience suggests that high-fiber foods may worsen intestinal symptoms during radiotherapy and in Sweden, several clinics recommend reducing or avoiding those kinds of foods (9). However, reduced intake of dietary fiber has not been shown in existing studies to provide any advantage compared to a diet with a higher fiber intake (10–13). A Cochrane review found that low-certainty evidence suggests that a high-fiber diet may in fact lead to a better outcome of intestinal health 1 year after radiation treatment (14). The result was mainly based on a study by Wedlake et al. where the high-fiber group had a target of ≥18 g non-starch polysaccharides per day (10). Previous pilot studies have shown that fiber supplements also can be beneficial for patients in reducing the incidence of diarrhea during radiotherapy (15, 16). Mechanisms for this effect could be that dietary fiber helps to preserve the two protective mucus layers and hinder or prevent starvation by supplying short-chain fatty acid to the colonocytes (17). Lack of mucus, as well as starvation of the colonocytes, may decrease the integrity of the epithelial barrier that separates pathogens in the lumen from the host (18). This may cause acute inflammation. This acute inflammation may in turn be followed by several different pathophysiological processes, including a self-propagating low-grade chronic inflammation. Data from well-powered randomized controlled trials are needed to provide an evidence base for a recommendation of a fiber-rich diet during pelvic radiotherapy.

To increase the evidence concerning advice on dietary fiber given to patients receiving pelvic radiotherapy, a randomized controlled intervention study, FIDURA, was planned. In this article, we present results from a preparatory study (hereafter called pre-study) in which we continuously tuned or refined the conditions during the course of the study in order to test and evaluate the logistics, procedures, and experiences of a dietary fiber intervention among patients undergoing radiotherapy to ensure feasibility and maximize compliance and completeness of the subsequent main study, the FIDURA-study.

## Materials and Methods

### Study Population

Inclusion criteria were women at any age diagnosed with gynecological cancer scheduled for pelvic radiotherapy with curative intention. The exclusion criteria were intestinal stoma or reservoir and difficulties in reading or understanding Swedish. The Swedish Ethical Review Authority approved the study (Approval Code: 803-18).

### Logistics

#### Inclusion Procedure

All patients, matching previously stated criteria, treated at the Oncology Clinic, Sahlgrenska University Hospital in Gothenburg, Sweden, from January 1, 2019, to August 31, 2020, were eligible for study inclusion. Treating physicians identified patients and an assistant nurse sent the patients an invitation letter. A dietitian from the study administration called them later on. During the phone call, patients had the opportunity to ask questions and to report if they were interested or not in participating.

#### Inclusion Meeting

We planned an inclusion meeting with interested patients in conjunction with a preparatory visit at the Radiotherapy Clinic around 2 weeks before radiotherapy was to start. The period of 2 weeks was chosen to adjust the patients to the increased dietary fiber intake before radiotherapy started (19). The inclusion meeting could also be held *via* telephone.

Patients were registered in a study-specific database and were automatically randomized to one of two groups, which—for participants included in the latter part of the pre-study (Participant Number 36-57)—indicated which intervention they would receive. Before or at the inclusion meeting, the patients were registered for the study online, where they gave their informed consent and received a study identification number and password for the website and a digital application for mobile phone or tablet (hereafter called application). The inclusion meeting also included the provision of overall study information and demonstrating the application to the patients. The patients filled out the first questionnaire and gave blood samples. We intended to measure patients' height and weight at the inclusion meeting, but to minimize the effort for the patients we changed to accepting self-reported measurements.

### Intervention

#### Psyllium Intervention

We discussed different types of fiber interventions and if a low-fiber group should have been included. Due to existing evidence, we concluded it would not be ethical to have a low-fiber intervention (10, 14, 17). Intake of psyllium had previously been shown to reduce the incidence and severity of radiation-induced diarrhea in patients during pelvic radiotherapy (15), and improvements of stool consistency and frequency in persons with fecal incontinence (20, 21). A few oncological clinics in Sweden had previously advised pelvic cancer patients to use psyllium as a supplement during their cancer therapy and had reported minimal adverse effects (9). Psyllium is rich in gel-forming soluble fibers, which are intermediately fermentable in the colon (22, 23). Psyllium increases the production of short-chain fatty acids such as butyrate, which is the main source of energy for the colonocytes (24, 25).

Psyllium husk was provided to participants in three different ways: powder, unblinded capsules (known to contain psyllium), and blinded capsules (unknown if they contained psyllium or placebo). Of those patients eating the psyllium powder, we let six participants try a low-fiber thickener as a placebo powder. We also let some patients try taking psyllium both in the form of powder and in capsules to see which one they preferred. For the psyllium powder, patients were instructed to eat 12 g [=3 teaspoons, which corresponds to 9 (78%) g of dietary fiber (26)], divided on two occasions per day. We recommended that the powder be mixed in beverages like water and juice or foods like porridge and yogurt. To provide the corresponding amount of psyllium powder, 12 g, in capsules (specially made for the study) was too much for patients to ingest daily (28 average-sized capsules). Therefore, in dialog with participants, we lowered the number of capsules to 15. Fifteen capsules contain a total of 6.5 g psyllium husk, which corresponds to 5.0 g of dietary fiber (26).

We gave patients instructions on how to ingest the powder or the capsules, including intake of extra fluid, and told them to gradually step up the intake during 1 week, and avoid intake with other medicines since psyllium might affect the absorption of those medicines (27).

#### Dietary Advice and Recipes

We designed advice concerning the choice of food items based on findings in our national Swedish survey including dietitians and nurses working with patients undergoing pelvic radiotherapy (9). We decided to encourage the patients as far as possible to eat a high-fiber diet that could be tolerated on an individual basis. In the first part of the pre-study, we formulated a daily goal for the participants to consume between 15 and 20 g of dietary fiber per day from the regular diet. Later on, we gave a more precise figure; we encouraged participants to consume at least 16 g of fiber per day. This amount was similar to the intakes reported by Wedlake et al. (10), where participants on average reached the intake of 16 g of dietary fiber per day at the end of radiotherapy—even if the goal was to reach at least 18 g per day.

To help the patients to achieve the goal of a specified daily dietary fiber intake, we created a list of fiber-containing food items and high-fiber recipes they were encouraged to follow. The recipes include easily prepared dishes, and these were created in collaboration with two award-winning chefs and experienced dietitians working with these patients.

### Data Collection

The day after the inclusion meeting, patients were instructed to collect their first fecal sample, start the fiber intervention, and begin to register information in the application twice a week. The participants registered food intake, frequency and consistency of stools, and other information in the application (Table 1). Patients were advised about taking the capsules until the follow-up appointment, about 1 month after radiotherapy. Fecal samples were collected on four more occasions−1 day before radiotherapy started, the last week of radiotherapy, 1 month after radiotherapy, and 1 year after radiotherapy. Blood samples were collected on three more occasions—in the middle of radiotherapy, 1 month after radiotherapy, and 1 year after radiotherapy. The primary outcomes of the planned main study will be obtained from the third blood sample 1 month after radiotherapy and from the questionnaire 1 year after radiotherapy.

**Table 1:** Questions, statements, and answering categories used in the application.

**Questions/statements**	**Answering categories**
Enter all toilet visits with stools you had during the current day (for example, Wednesday). Enter the consistency according to the Bristol scale below. When you have completed the type of texture, click “Done” at the bottom of the page. Repeat this for every toilet visit.	Bristol Stool Form Scale: •Type 1 •Type 2 •Type 3 •Type 4 •Type 5 •Type 6 •Type 7 •I had no toilet visit with stool today
What gas and bowel regulators, such as stopping agents or laxatives, have you taken in the past week? If you have taken more medicines, you must register them one at a time.	•I have not taken gas and bowel regulators this week •Dimetikon Meda •Minifom •Dimor •Loperamide •Imodium •Vi-Siblin •Lunelax •Inolaxol •Cilaxoral •Movicol •Other
What dose of gas and bowel regulators have you approximately taken this week?	•1–14 (ca 1–2 per day) •15–28 (ca 2–4 per day) •29–42 (ca 4–6 per day) •More than 43 (more than 6 per day)
How troublesome has your bloating, abdominal tenderness, or pain been the past week?	•Not relevant, I have not been bloated or had abdominal tenderness or pain •Not at all troublesome •A bit troublesome •Moderate troublesome •Very troublesome
How troublesome has your flatulence been this past week?	•Not relevant, I have not had any flatulence •Not at all troublesome •A bit troublesome •Moderate troublesome •Very troublesome
How many FIDURA capsules have you taken per day the past week?	•No capsules •About 1–5 capsules per day •About 6–10 capsules per day •About 15 capsules per day •About 16–20 capsules per day

We created a specific website for the study. On this website, patients could find all the study information and register for the study. The patients who lacked digital equipment, or who did not want to get the information online, got printed paper versions. If patients wanted, they could get automatic SMS reminders to take blood- and fecal samples, fill in the questionnaires, and register information in the application. We controlled the reminders from the study-specific database. We also programmed the database to remind us about specific dates when we would book blood samples and contact the patients for follow-ups, etc.

**Figure 1:**
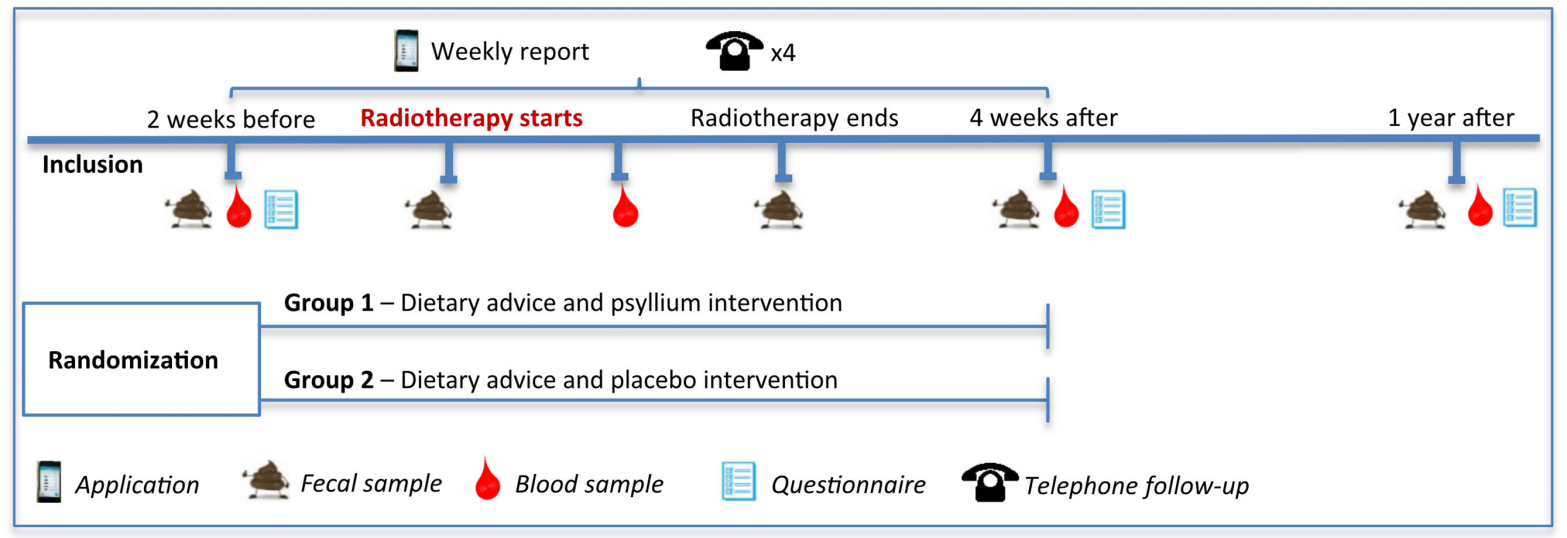
The final study design of the planned main study.

We made repeated telephone follow-ups to give patients individual dietary advice and ensure they had not experienced aggravated symptoms from the intervention. We scheduled four follow-ups during the intervention. The study design for the final large-scale data collection that we decided on after this pre-study is shown in Figure 1.

#### The Application

##### The Measure of Dietary Fiber Intake

The development and description of the assessment of dietary fiber intake using the application have been described in detail elsewhere (28). Briefly, the application enables reporting intake of common high-fiber foods and corresponding foods with a low amount or no fiber (e.g., whole grain pasta and white pasta). Patients registered foods in predetermined measurements (e.g., numbers, deciliters, and slices). In our validation study, the dietary fiber intakes estimated from reports given by use of the application were compared to dietary records and found to provide accurate estimations under common conditions (28). During the pre-study, we asked the participants if certain foods were lacking in the application or if additional technical functions in the application were needed. We then made any relevant changes in the application.

##### Other Features in the Application

Except for the food registration, the use of the application also included reporting daily stools and a weekly report of medicines, flatulence, abdominal pain, and intake of study capsules (see Table 1). The application registered stool frequency in combination with stool consistency using the Bristol Stool Form Scale (29). For participants to register in the application every day during a period of at least 10 weeks did not seem reasonable since it would likely increase the risk of study dropouts. Therefore, we decided that once-a-week reporting was a reasonable compromise between compliance and representativeness of the intake estimations. If the patients wanted to, they could register foods and stools more than one time per week. We decided to instruct patients to register foods and stools on Wednesdays because we wanted a day in the middle of the week. For the rest of the variables in the application, for example medicines, we decided to instruct them to register on Sundays, to report the situation from the past week.

The application was also a tool that could help patients see how much fiber they had consumed per day and to see statistics on their stool habits. For patients who lack a smartphone, we created a paper version. The printed paper version could of course not give the same feedback about the amount of fiber they ate per day; therefore, for those who used the paper version, we offered to calculate the intake of fiber for them.

#### Questionnaires

To estimate the entire dietary intake at different time points during the study we used a food frequency questionnaire from a previous study with prostate cancer patients as a starting point (30). We used the same 14 food group categories (sandwiches; porridge and breakfast cereals; nuts and seeds; drinks; vegetables and fruits; meat and meat products; fish and shellfish; egg dishes and vegetarian options; potatoes, pasta, rice, and grains; fast food; fat; sauce and dressing; sweets, snacks, and dessert cheese) which included several food items and complex dishes. We used the same arrangement of the questions that first included how often (number of times daily, weekly, monthly, or never) a food group or a dish had been eaten, and then a sum of ten would be distributed on specific food items included in the food group or ingredients that the dish can be made of (e.g., wheat, rye, and oats). Standard portions are based on information from the Swedish National Food Agency (31) and the content of macronutrients and micronutrients is calculated using the database from the Swedish National Food Agency version 2020-08-10 (26).

The original questionnaire also included questions about physical activity, intake of food supplements, tobacco use, medications including the use of antibiotics, other diseases, socio-economic parameters, and anthropometric measurements. We included most of those questions with some adjustments; the questions were for example adapted to patients receiving pelvic radiotherapy. We added more high-fiber foods and questions including intake of probiotics, how often different intestinal symptoms have been experienced, as well as if specific foods or physical activities have improved or worsened by these intestinal symptoms. The questions about intestinal symptoms are validated and have been used in earlier studies (1, 32, 33). Possible confounding factors (e.g., diseases, medicines, etc.) were also included in the questionnaire.

We planned for patients to answer the questionnaire at three time points: 2 weeks before radiotherapy started and 1 month and 1 year after the end of radiotherapy. We designed the questionnaires to be quite similar and adjusted them as needed according to their time points. For the second questionnaire, we designed a question about the preferred source of fiber: from diet, supplements, or medicines. For the second and third questionnaires, we designed questions on whether patients avoid any foods or physical activities due to their pelvic radiotherapy.

We shortened the part of dietary questions in the second questionnaire, to cover only the dietary change during radiotherapy. For example, one question included if patients had eaten less-, no change-, or had eaten more of different food groups (e.g., bread, vegetables, and sweets). We decided it was not necessary to cover the whole diet a second time, to reduce the effort and time needed for the patients to provide the information. Due to the blinded part in the main study, we added a question if the patients believed they had gotten fiber in their capsules or not, or if they did not know.

#### Blood Samples

Our plan was to collect blood samples on five occasions during the study but to reduce the burden on the participants this was changed to four times. The blood samples were drawn in connection with clinical visits and if possible, together with another clinical sampling.

#### Fecal Samples

We decided to collect fecal samples on five occasions during the study and planned that the patients would send them by mail. However, to improve the quality of the fecal samples and to prevent markers from being affected, we decided that patients should be asked to freeze the fecal samples at home and bring them frozen at ordinary clinical appointments. If this was not possible, patients were advised to send the samples by mail.

### Statistical Analyzes

Statistical analyses were used to guide our approaches rather than to evaluate the effects. We used the Mann-Whitney *U*-Test, due to non-normally distributed data, to examine if there was any difference in age between patients who used the digital versions and the paper versions of the application and the questionnaires. We divided the participants into five groups according to the time point when they had been included in the study to investigate if the participation rate for the third blood sample and the baseline questionnaire had changed during the pre-study. The Kruskal-Wallis-test was used to investigate if the length of the intake periods differed between the different psyllium- and placebo interventions. A *post-hoc* test was used to investigate between which groups a possible difference was present. A linear mixed-effect model was used to analyze the total fiber intake registered by the application to handle the individual level clustering present in the data. Analyses excluding outliers and participants with 2 or fewer days of registration were also performed. Statistical analyzes were executed using IBM SPSS Statistics 26 and RStudio Version 1.1.463.

## Results and Discussion

### Inclusion Procedure

We discovered that patients often declined study participation at the first phone call with the argument that they were overwhelmed with information. Therefore, we tried a second, shorter, version of the invitation letter, cutting out about half of the initial text. The invitation letter was first sent in the same envelope as a call for an appointment of the patient with the treating physician at the Oncology Clinic. We realized the patients often had not noticed our letter; they had stopped reading after understanding when their appointment was scheduled. We tested sending the study letter in a separate envelope, and this gave better results. This could be explained by the fact that the letter had disappeared among all other information that patients received about the radiotherapy from the responsible clinic. Instead of the phone call, we also tested asking the patients in real life, by a dietitian from the study secretariat, about study participation in connection with their appointment with a physician at the Oncology Clinic. This worked out well but it was very time-consuming and thus was found not to be a desirable approach for a larger study.

**Figure 2:**
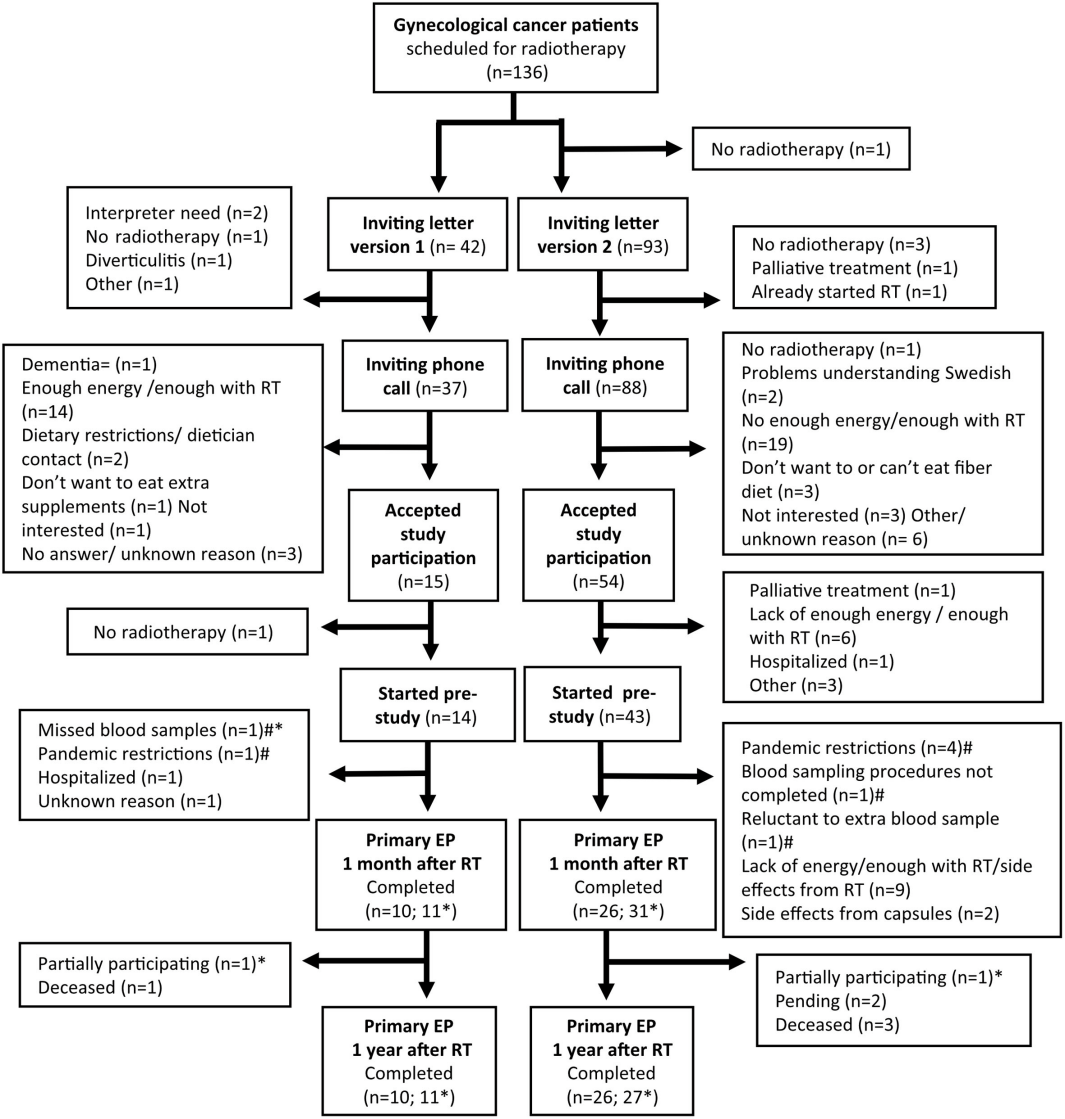
Flowchart of the preparatory study. RT, Radiotherapy; EP, endpoint. The third blood sample was the primary endpoint 1 month after radiotherapy and the third questionnaire was the primary endpoint 1 year after radiotherapy. *Completed either blood sample, fecal sample, or questionnaire. ^#^Continuing preparatory study.

Figure 2 shows a flowchart of the pre-study. One hundred thirty-six women with gynecological cancer were scheduled for curative pelvic radiotherapy and all—except one patient with canceled radiotherapy—were sent an invitation letter for participation in the study. Forty-two patients received the longer version of the letter and 93 patients received the shorter version of the letter. We called 125 of the patients, and 41% of those who received the long letter and 61% of those who received the short letter orally accepted study participation. It is challenging to balance the amount of information given to patients in a study and we wanted to ensure the patients got sufficient information that they could comfortably read.

A total of 57 patients started the pre-study. The most common reason for declining participation was that they found radiotherapy sufficiently challenging and did not have enough energy to participate in the research. A comparable reason to decline participation was the most common in a previous study with a similar population (34). The most common reason for exclusion in our study was canceled radiotherapy. Thirty-six patients completed the blood sampling (primary endpoint) 1 month after the end of radiotherapy, and 42 patients completed at least one part of the follow-up (Figure 2). To this date, 36 patients have completed the questionnaire (primary endpoint) 1 year after the end of radiotherapy; reports from two patients are still pending. The most common reason for dropouts during the study was the same as for declining participation before the study started, often due to side effects related to the radiotherapy. All dropouts occurred between the study inclusion and the first follow-up and the only reason yet, for not completing the second follow-up, has been if a patient had died. The median time from the dropout date to the date when radiotherapy started was 0 days (range −14–49). A higher proportion of patients accepted study participation after we shortened the letter, but we also had a higher dropout rate of those starting the study. Radiotherapy, sometimes in combination with chemotherapy, is a challenging period for patients. The treatment is time- and energy-consuming and causes side effects like fatigue, nausea, and diarrhea (35, 36). Several of the patients were already exhausted before starting the radiotherapy and experienced fatigue due to their cancer disease and earlier treatments. A qualitative study by Jakobsson et al. showed that gastrointestinal symptoms and pain caused most distress in patients during pelvic radiotherapy (37). This is a probable cause of why all our dropouts occurred within the period of 1 month after the end of radiotherapy.

### Patients Characteristics

Selected characteristics and demographics of the participants are shown in Table 2. The patients who started the pre-study had a median age of 69 years (range 34–83), endometrial cancer was the most common diagnosis, the median external radiation dose was 56 Gy (range 33–77), and the median number of fractions was 28 (range 11–35). Nineteen percent of the patients had brachytherapy (2 × 6 Gy) during radiotherapy and 46% of the patients had chemotherapy before radiotherapy and 37% during radiotherapy. Most patients (62%) had never smoked, had a low physical activity (53%), were married or had a partner (51%), and had a higher education than primary and middle school or equivalent (85%).

**Table 2:** Baseline characteristics of the participants in the preparatory study.

	**Median**	**IQR**	**Range**
Age at inclusion, years (*n* = 57)	69	21	34–83
External radiation dose, Gy (*n* = 57)	56	19	33–77
Fractions, number (*n* = 57)	28	4	11–35
Length of radiotherapy, days (*n* = 57)	39	8	14–50
	***n***	**%**	
**Sex (*****n*** **=** **57)**
Female	57	100	
**Gynecological site (*****n*** **=** **57)**
Endometrial	27	47	
Cervical	21	37	
Vulvar	7	12	
Vaginal	1	2	
Tubar	1	2	
**Chemotherapy (*****n*** **=** **57)**
Before radiotherapy	26	46	
During radiotherapy	21	37	
**Brachytherapy (*****n*** **=** **57)**	11	19	
**Smoking (*****n*** **=** **47)**
Never smoked	29	62	
Previously smoker	14	30	
Current smoker	4	9	
**Physical activity (*****n*** **=** 47)^*^
Low	25	53	
Moderate	10	21	
High	12	26	
**Education (*****n*** **=** **47)**
Primary and middle school or equivalent	7	15	
High school, technical high school, or equivalent	20	43	
University or college	20	43	
**Civil state (*****n*** **=** **47)**
Married/partner	24	51	
Unmarried/widower/divorced/living apart	23	49	

*IQR, Interquartile range. Baseline characteristics were collected from the first questionnaire or patients' medical records. The percentages were rounded to the nearest integer*.

* *The level of physical activity was calculated from two questions in the baseline questionnaire corresponding to the activity the past week and the answers were assigned specific points. “How much time during the past week did you devote to physical training that resulted in you being out of breath, for example, running, gym classes, ball or team sports?”. None (0 points), up to 0.5 h (2 points), 0.5–1 h (6 points), 1–1.5 h (12 points), 1.5–2 h (18 points), 2.5–5 h (30 points), more than 5 h (60 points). “How much time during the past week did you devote to daily physical activity, for example walking, bicycling, or working in the garden?”. None (0 points), up to 0.5 h (1 point), 0.5–1 h (3 points), 1–1.5 h (6 points), 1.5–2 h (9 points), 2.5–5 h (15 points), more than 5 h (30 points). Low activity level was considered as <12 points, moderate activity level as 12–29 points, and high activity level as ≥30 points*.

### Logistics

Almost all patients wanted to meet in person at the inclusion meeting, even if the visit at the clinic became longer. During the pandemic of COVID-19, all physical meetings in the study were canceled and all inclusion meetings were held by telephone and patients were very thankful for this. If possible, we let patients read the study information and start to fill out the first questionnaire at home before the inclusion meeting, which eased the burden they faced at the meeting. We tried to customize the participation in the study based on the patients' abilities and by so doing, decrease the dropout rate. We started to inform patients at an early stage in which parts of the study were most important to participate in. If they experienced participation in the study to be too much for them to handle, we asked if they could participate in fewer parts of the study, for example only eat the capsules and leave the blood samples. For some, this helped but for others, it seemed likely that they had already decided to leave the study. We experienced that pausing the participation for a few weeks, during patients' toughest periods, could be effective in helping to keep the patients in the study. We asked the patients if they wanted the study material in printed paper form even if all information was available on the website, and most patients preferred the study material in printed paper form.

### Study Intervention

#### Psyllium Intervention

We decided to use the powder of psyllium husk from Finax® other brands were more voluminous and using them would have forced the patients to eat large volumes. Table 3 shows the 52 participants who started the psyllium- and placebo intervention, what form of psyllium or placebo they received, and the length of the intake period. We gave the first participants psyllium powder to examine how they experienced eating it during the radiotherapy. Due to the water-soluble and gel-forming characteristics of psyllium, it quickly becomes thick and gets a texture similar to a thick jelly. These characteristics were not a problem for patients at the beginning of radiotherapy. The patients who became nauseous during radiotherapy, mainly those receiving chemotherapy, had a huge problem with the texture and the flavor of the psyllium powder. Those patients could not continue to eat the powder in the last weeks of radiotherapy because they related the psyllium to the nausea they were experiencing. This increased the risk of poor compliance. The patients also struggled to eat the low-fiber thickener. We did not consider it ethical to continue this path.

**Table 3:** Psyllium- and placebo interventions and length of the intake period (*n* = 52).

**Psyllium- and placebo intervention**	**Intake period ≤1 week *n*/tot *n* (%)**	**Intake period >1–9 weeks *n*/tot *n* (%)**	**Intake period ≥10 weeks *n*/tot *n* (%)**
Unblinded powder	1/7 (14)	5/7 (71)	1/7 (14)
Combination of unblinded powder/capsules	0/6 (0)	0/6 (0)	6/6 (100)
Unblinded capsules	2/19 (11)	6/19 (32)	11/19 (58)
Blinded capsules	4/20 (20)	8/20 (40)	8/20 (40)

*The percentages were rounded to the nearest integer. The Kruskal-Wallis test showed a statistically significant difference in the lengths of the intake periods between the psyllium- and placebo interventions (p = 0.026). The post-hoc test suggested that patients' intake of the combination of unblinded powder and capsules was longer than the intake of the unblinded powder (p = 0.057; adjusted for multiple comparisons). The adjusted results for the remaining interventions were unblinded powder—unblinded capsules p = 0.90; unblinded powder—blinded capsules p = 1.00; combination of unblinded powder/capsules—unblinded capsules p = 0.92; combination of unblinded powder/capsules—blinded capsules p = 0.12; unblinded capsules—blinded capsules p = 1.00*.

About half of the patients preferred the capsules and the other half preferred the powder. Most patients who did not feel nauseously experienced taking the powder as feeling more natural than taking the capsules. It would be difficult to let the participants choose what form they want in the main study—the amount of fiber will then differ. We, therefore, decided to use capsules either with psyllium or placebo (maltodextrin, see below), which made it possible for us to triple-blind- and decrease potential bias in the main study. This might result in a small loss of participants preferring psyllium powder. Instead of the thickener, we chose maltodextrin as a placebo because it does not contain dietary fiber and has been used in other studies (38). Most patients thought that they could manage to take 15 capsules, divided into three time intervals per day. Before the blinded capsules were ready, all participants received either psyllium powder or unblinded capsules, regardless of which group they had been randomized to. Most patients (*n* = 20) received blinded capsules and 20% interrupted this intervention within a week. Study dropouts, bowel-related problems, and difficulties swallowing capsules were the most common reasons for early termination of the intervention. All patients (*n* = 6) who received a combination of unblinded psyllium-husk powder and capsules completed the intervention. Of the dropouts, one participant received unblinded powder, five participants fiber capsules, and two blinded capsules. The Kruskal-Wallis-test showed a statistically significant difference in the lengths of the intake periods between the psyllium- and placebo interventions (*p* = 0.026; Table 3). The *post-hoc* test suggested that patients' intake of the combination of unblinded powder and capsules was longer than the intake of the unblinded powder (*p* = 0.057; adjusted for multiple comparisons) (Table 3). There seems to be considerable variation in the individual preferences of patients. Therefore, patients in clinical practice may be offered both powder and capsules, if the intervention turns out to be effective.

Results from the second questionnaire showed that 25% of the patients receiving the blinded capsules believed they had fiber capsules, 5% believed they had placebo capsules, 35% did not know, 20% answered they had not eaten the capsules, and 15% did not fill out the second questionnaire. Based on these varying answers the blinding of the capsules was working.

#### Dietary Advice and Recipes

We changed the daily goal from 15 to 20 g to at least 16 g of dietary fiber per day from the regular diet, to change to a more precise cut-off value for patients to compare with values in the application. Some of the patients appreciated getting dietary advice and recipes at the inclusion meeting; others thought they had already received enough information. A few patients wished they could be given more recipes, so we added some additional recipes. During the follow-ups, we discovered that in reality, few patients had used the recipes since they already were very much occupied and suffered from fatigue related to the treatment.

### The Application

In total, 34 patients started using the application or the paper version, and 62% of them completed the registration of at least 10 weeks (Table 4). Twenty-one percent of the patients declined using the application or the paper version, mainly because they felt that use required too much effort together with the other parts of the study. Twelve patients used the paper version, and the main reason was a lack of a smartphone, but some were inexperienced with downloading and using applications. There was a statistically significant difference in the distributions of ages between those using the application (median age 55 years) and those using the paper version (median age 75.5 years) (*p* < 0.001, Mann-Whitney *U*-test). We added some requested foods (for example roasted soybean snacks) and useful functions (for example the amount of fiber shown directly when the food item was chosen). We also added the dishes from our recipes to the application to further ease the registration of fiber-containing foods.

**Table 4:** Percentage of patients delivering information on dietary fiber intake and acute side effects *via* an application (mobile phone or tablet) or paper version.

**Started application or paper version *n*/tot *n* (%)**	**Declining application and paper version *n*/tot *n* (%)**	Not applicable^*^n/tot *n* (%)	**Finished application or paper version^#^ *n*/tot *n* (%)**	**Used the application *n*/tot *n* (%)**	**Used the paper version *n*/tot *n* (%)**
34/57 (60%)	12/57 (21%)	11/57 (19%)	21/34 (62%)	22/34 (65%)	12/34 (35%)

* *Not applicable was defined as study dropouts affecting participation in the application and application logistics not finished at the time for study inclusion*.

# *Ten weeks of registration was defined as finishing the application*.

**Figure 3:**
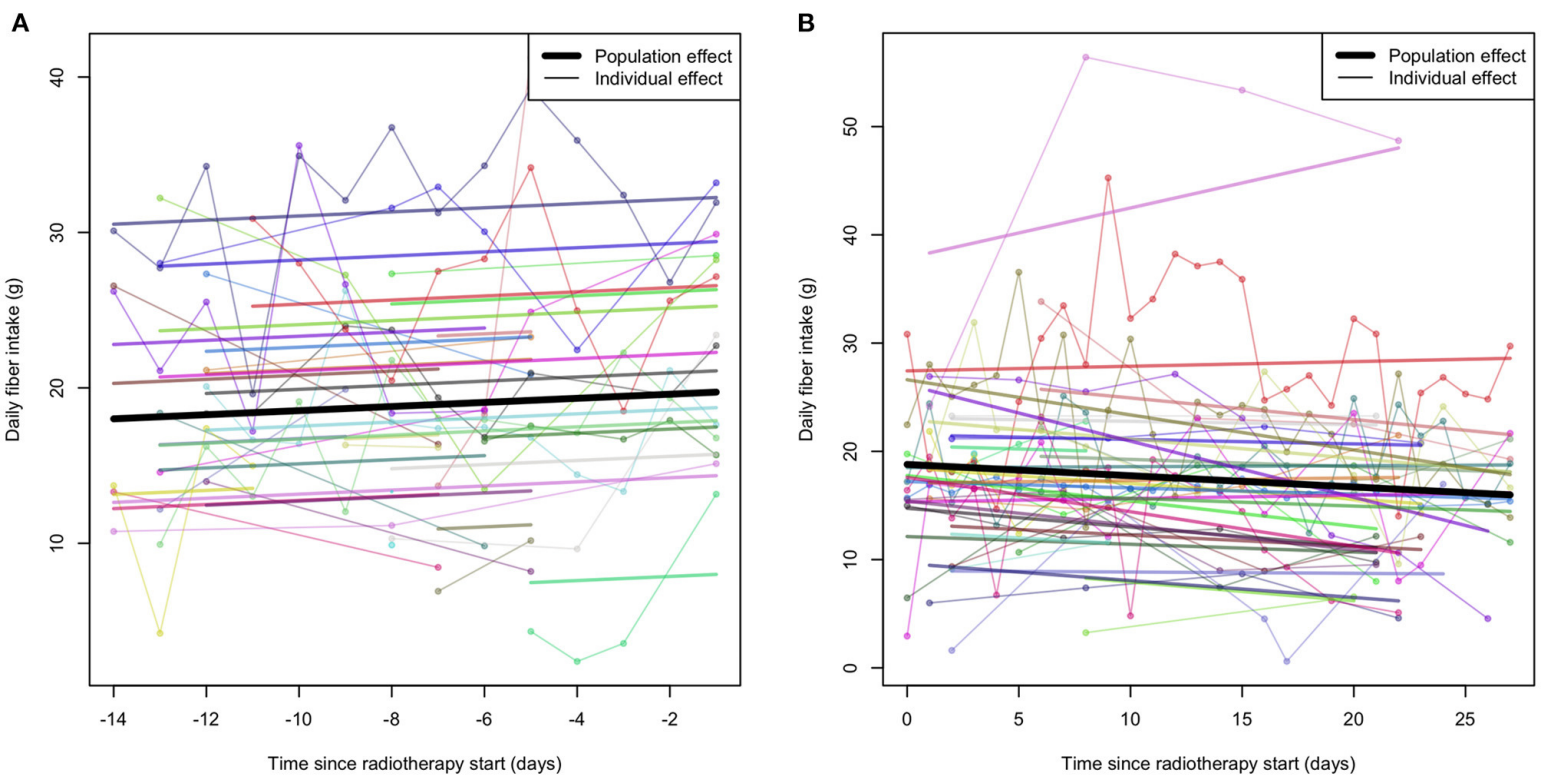
Individual total intake of dietary fiber (g) per day registered by the application. Regression lines were estimated using linear mixed-effects models allowing for random intercepts and slopes at the individual level. **(A)** Shows the intake before the start of the radiotherapy (*n* = 24). **(B)** Shows the intake after the start of the radiotherapy (*n* = 27).

The median intake of dietary fiber 2 weeks before radiotherapy started was 20.0 g/day (*n* = 22; range 4.2–38.0) and the median intake 4 weeks after radiotherapy started was 16.2 g/day (*n* = 24; range 1.3–20.6) (Figure 3). The fixed effect including all participants 2 weeks before the radiotherapy to the start were not statistically significantly different from non-zero (Figure 3A; β = 0.132; *p* = 0.316). The analysis excluding participants with two or fewer registration days did not converge, but excluding outliers did not substantially alter the results (*n* = 24; β = 0.107; *p* = 0.437). The result of the fixed effect including all participants from the start of the radiotherapy to 4 weeks after were not statistically significant (Figure 3B; β = −0.104; *p* = 0.142). The result became statistically significant when we excluded outliers and participants with few registrations (*n* = 22; β = −0.137; *p* = 0.0365). The median number of registrations per participant during the study was 37.5 times (range 1–68). A statistical analysis plan should include information about how to handle missing information.

The individual variation in fiber intake was high, which is also in line with what is known for the general population of Swedish women (39). Patients in the study of Wedlake et al. (10) decreased their mean intake of dietary fiber from 17.1 g per day at the start of radiotherapy to 15.7 g per day at the end of radiotherapy. Our patients showed a trend of decreasing their intake during radiotherapy (Figure 3B), but the median intake exceeded, in any case, our fiber goal of 16 g of fiber per day. The decreased intake of fiber could be a result of incomplete reporting in the application or a truly decreased fiber intake. Both incomplete reporting and a truly decreased intake may be due to side effects from the radiotherapy. We experienced that the application is a good tool to give a response to patients' intake of dietary fiber. Developing the application was time-consuming but made it possible to give the patients information in return, which a dietary record does not. Some patients were unable to complete the registration of the application the entire period, others registered more often than we requested.

### Questionnaires

Eighty-two percent of the patients completed the first questionnaire, and most patients used the web version (Table 5). The median response time was 50 min (range 10–1440) for patients to complete the first questionnaire. Seventy percent completed the second questionnaire and 65% have to this date, finished the third questionnaire. Half of the patients used the web version and the other half the paper version for the second questionnaire and most patients (53%) chose the paper version for the third questionnaire. For all questionnaires, those choosing to use the paper version were older than those choosing the web version (first questionnaire *p* = 0.038; second questionnaire *p* < 0.001; third questionnaire *p* = < 0.001, The Mann-Whitney *U*-test was consistently used) (Table 5). Hutchesson et al. showed that online food records on computers or smartphones were more acceptable than paper-based food records by young women (40). Patients mainly declined the questionnaires to minimize the burden of the study. The first questionnaire had the highest participation rate. Our impression is that the abbreviated version and the time point for the second questionnaire 1 month after the end of radiotherapy promoted a high participation rate. Even if some patients stopped providing information in the application during the radiotherapy, side effects often had been relieved a few weeks after the end of radiotherapy and they were able to complete the second questionnaire. To date, a few patients have not reached the time for the third questionnaire, but the participation rate so far has been promising.

**Table 5:** Participation in the questionnaires.

	**Questionnaire 1**	**Questionnaire 2**	**Questionnaire 3**
**Completed** *n*/tot *n* (%)	47/57 (82)	40/57 (70)	36/55 (65)^#^
**Not applicable** *n*/tot *n* (%)	5/57 (9)	14/57 (25)	17/44 (39)^#^
**Declined** *n*/tot *n* (%)	5/57 (9)	3/57 (5)	2/44 (5)^#^
**Web** *n*/tot *n* (%)	24/42 (57)^*^	20/40 (50)	17/36 (47)^#^
**Paper** *n*/tot *n* (%)	18/42 (43)^*^	20/40 (50)	19/36 (53)^#^
**Median age web version** (range)	55.5 (34–79)^*^	55 (34–76)	55 (34–73)^#^
**Median age paper version** (range)	72 (49–83)^*^	72 (49–83)	71 (49–83)^#^
***P*** **-value**	0.038^¤^	<0.001^¤^	<0.001^¤^

*The percentages were rounded to the nearest integer. Not applicable was defined as study dropouts and deceased patients*.

* *Five patients were not included because they were recruited before the web version was finished*.

# *Two pending patients were not included*.

¤ *The Mann-Whitney U-test was used to calculate the difference in age between those using the web version and the paper version of the questionnaires*.

**Figure 4:**
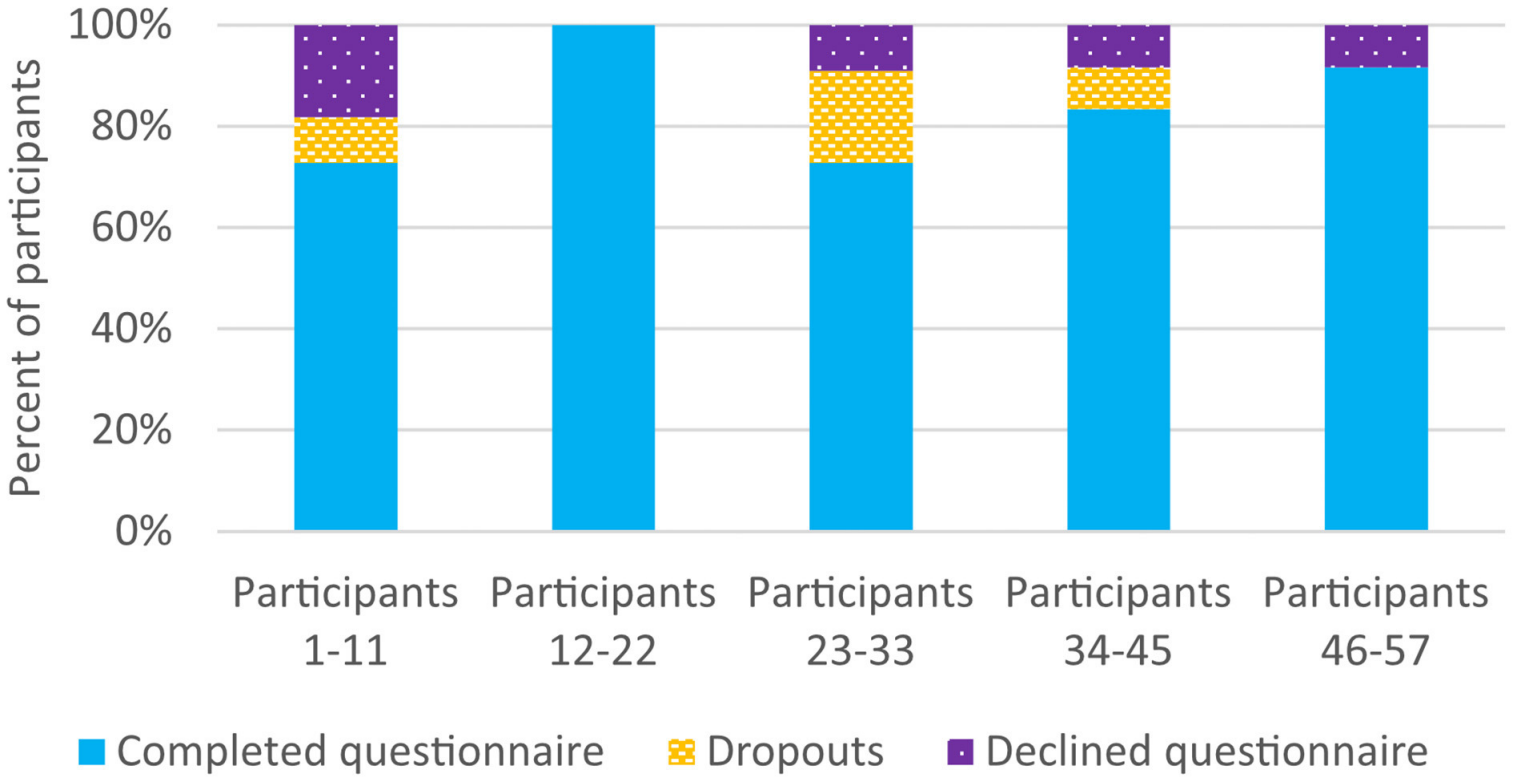
Participation in the baseline questionnaire, divided into five groups according to the inclusion order in the preparatory study.

The proportion of patients who completed the baseline questionnaire, divided into five groups according to the inclusion order in the pre-study is shown in Figure 4. The participation rate exceeded 70% for all groups. Most participants declined the baseline questionnaire during participants 1–11 and the highest dropout rate was during participants 23–33, possibly due to the ongoing pandemic of COVID-19.

#### Nutritional Data and Body Mass Index

Table 6 shows the nutritional intake and BMI at baseline. Most patients were obese (51%), the reported mean daily energy intake was 1,804 kcal (range 835–3,435) and the daily intake of fiber was 23 g (range 6–50). The estimated energy intake is consistent with the average energy intake for the Swedish female population (1,774 kcal) in the national survey Riksmaten (39). We identified a large spread between patients' intake of dietary fiber with both the first questionnaire and the application. The fiber intake among patients reported in the application before radiotherapy was similar to the fiber intake among the Swedish women population (31–80 years, intake of 18.5–20 g/day) (39), even if the reported fiber intake from the questionnaire was slightly higher. Few have investigated dietary fiber intake during pelvic radiotherapy, but the fiber intake in our study was similar to amounts in earlier studies (10, 12). BMI was based on self-reported measurements, which may differ to values reported by a healthcare professional.

**Table 6:** Baseline nutritional data and Body Mass Index from 47 of the participants, calculated from the first questionnaire.

	**Mean**	**SEM**	**Range**
Energy, kcal/d	1,804	72	835–3,435
Carbohydrates, g/d	192	8	55–315
Protein, g/d	76	3	28–197
Fat, g/d	74	4	26–130
Fiber, g/d	23	1	6–50
**BMI (kg/m** ^**2**^ )^*^	***n***	**%**	
Underweight (<18.5)	1	2	
Normal weight (18.5–24.9)	9	19	
Overweight (25–29.9)	13	28	
Obese (>30)	24	51	

*SEM, Standard error of the mean*.

* *BMI was based on self-reported height and weight in the first questionnaire*.

### Blood- and Fecal Samples

**Figure 5:**
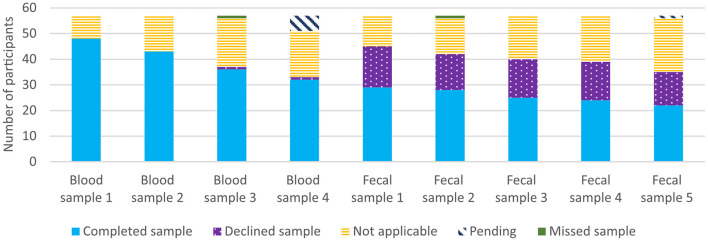
Participation in blood- and fecal sampling for all 57 participants. Not applicable was defined as missed samples due to study dropouts, deceased patients, a late inclusion in the study, sampling procedures not completed, and canceled clinical visits.

Eighty-four percent of the patients provided us with the first blood sample, 75% the second, 63% the third, and 63% the fourth (excluding six pending patients) (Figure 5). Our impression is that patients experienced that the blood sampling required little effort because they are accustomed to leaving blood samples in clinical practice. The patients were grateful when the blood samples were collected at the same time as other blood samples or other clinical visits. Fifty-one percent of the patients collected the first fecal sample, 49% the second, 44% the third, 42% the fourth, and 39% the fifth (excluding one pending patient) (Figure 5). The most common reason to decline providing the fecal samples was to minimize the burden of study participation—we prioritized the blood samples instead. This decision will decrease the problems with the loss to follow-up but was also reflected in a decreased participation rate in providing the fecal samples for patients recruited in the latter part of the pre-study (data not shown). Even if fecal sampling can be experienced as embarrassing (41) the declined proportion of fecal sampling did not increase over time, which suggests most patients did not disapprove of collecting them. However, some patients made clear that they did not want to collect fecal samples. Wedlake et al. only received fecal samples, at both baseline and endpoint, from 41 of 166 participants, which reduced the statistical power when fecal short-chain fatty acid concentrations were compared between groups (10).

The average time to send the fecal samples by mail was 4.4 days (range 3–10). We changed the address where patients sent the fecal samples and the average delivery time decreased to 1.7 days (range 1–3). By changing to a more specific address, the within-University delay could be shortened. The varying and sometimes delayed delivery times for the fecal samples sent by mail will affect the sample quality, and this needs to be taken into account when analyzing various markers (42).

**Figure 6:**
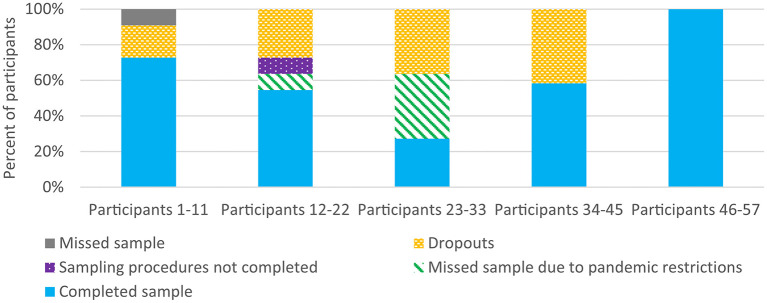
Participation in the third blood sample (primary endpoint), divided into five groups according to the inclusion order in the preparatory study.

Figure 6 shows the proportion of patients who provided the third blood sample, divided into five groups according to the inclusion order in the pre-study. The last 12 patients had the highest proportion of completed samples. A high proportion of participants 23–33 missed providing their samples due to COVID-19 restrictions. We had to reschedule taking some of the blood samples during the pandemic period and we established new logistics working during these new circumstances. The lower participation rate at the beginning of this pre-study we believe was due to a higher burden put on subjects due to more intensive samplings and investigations than other studies. Similar studies have reported participation rates in follow-ups of 96% at the end of radiotherapy, 85% at 8 weeks after baseline, and 76%, respectively, 82% at 1 year after radiotherapy (10, 13). It is probable that our adjustments during the inclusion period had an effect and resulted in fewer dropouts of participants recruited at the end of the pre-study compared to those participants recruited at the beginning. The pandemic may also have affected the response and dropout rate during this study.

### Follow-Ups

The telephone follow-ups were a great opportunity for us to collect information about patients' symptoms, to get feedback about the study, and support the patients. The four scheduled follow-ups were often enough, we did not experience that we contacted the patients too often nor too seldom. The phone calls lasted ~15 min. Additional shorter phone calls or text messages were often necessary to, for example, update missing information in the questionnaires or find out exact times to meet them at the hospital to pick up their fecal samples. We experienced positive reactions from the patients to have someone regularly following their well-being during the radiotherapy.

## Conclusion and Implication

Concise and relevant information provided to patients at the right time was crucial to achieving an acceptable participation rate and compliance in the study. The most common reasons for declining participation or dropping out were the expected burden of radiotherapy or acute side effects. Tailoring the ambition level to each patient concerning the collection of data beyond the primary endpoints was an important strategy to keep the dropout rate at an acceptable level. Using blinded psyllium capsules rather than a powder will decrease the risk of bias and outweighs any potential risk of lower compliance for participants. Using capsules makes it more easily feasible to compare results between studies in the future as data can be standardized. Among the 57 patients who started the study, the participation rate in the primary outcomes was 63% (blood sample) and 65% (questionnaire). Thirty-nine to 51% collected the fecal samples at five different time points. We improved the logistics and for the last 12 included, the participation rate was 100% for one of the primary outcomes and 92% for the baseline questionnaire. Challenges included side effects from radiotherapy influencing the motivation to participate and restrictions for the COVID-19 pandemic that hindered physical meetings with the patients. Encouraged by the high response rate among the last 12 patients in the pre-study, we have started the main study with the logistics used among those participants.

## Data Availability Statement

The raw data supporting the conclusions of this article will be made available by the authors, without undue reservation.

## Ethics Statement

The studies involving human participants were reviewed and approved by Swedish Ethical Review Authority. The patients/participants provided their written informed consent to participate in this study.

## Author Contributions

RA, CB, FS, GS, and MH contributed to the conception and design of the study. RA and IS collected the data. RA and VS performed the statistical analysis. RA wrote the first draft of the manuscript. All authors contributed to the interpretation of the data, manuscript revision, read, and approved the submitted version.

## Funding

This research received funding from the Swedish Cancer Society (Dnr 2018-656), Stiftelsen Jubileumsklinikens Forskningsfond mot Cancer (Dnr 2018:194), Sjöberg Foundation (Dnr 220-01-07-02), the LUA/ALF-agreement in West of Sweden Health Care Region (Dnr ALFGBG-727821), the ALF-agreement (Dnr ALFGBG-926421), Ekhaga Foundation (Dnr: 2018-18), and Swedish Research Council (Dnr 2018-03228).

## Conflict of Interest

The authors declare that the research was conducted in the absence of any commercial or financial relationships that could be construed as a potential conflict of interest.

## Publisher's Note

All claims expressed in this article are solely those of the authors and do not necessarily represent those of their affiliated organizations, or those of the publisher, the editors and the reviewers. Any product that may be evaluated in this article, or claim that may be made by its manufacturer, is not guaranteed or endorsed by the publisher.
